# Multidimensionality and scale in a landscape ethnoecological partitioning of a mountainous landscape (Gyimes, Eastern Carpathians, Romania)

**DOI:** 10.1186/1746-4269-9-11

**Published:** 2013-02-06

**Authors:** Dániel Babai, Zsolt Molnár

**Affiliations:** 1 Institute of Ethnology, Centre for the Humanities, Hungarian Academy of Sciences, H-1014 Budapest, Országház u. 30, Hungary; 2 Institute of Ecology and Botany, Centre for Ecological Research, Hungarian Academy of Sciences, H-2163 Vácrátót, Alkotmány u. 2–4, Hungary

**Keywords:** Ecological anthropology, Folk habitat, Traditional ecological knowledge, Nature conservation, Phytosociology, Mountain hay meadows

## Abstract

**Background:**

Traditional habitat knowledge is an understudied part of traditional knowledge. Though the number of studies increased world-wide in the last decade, this knowledge is still rarely studied in Europe. We document the habitat vocabulary used by Csángó people, and determine features they used to name and describe these categories.

**Study area and methods:**

Csángó people live in Gyimes (Carpathians, Romania). The area is dominated by coniferous forests, hay meadows and pastures. Animal husbandry is the main source of living. Data on the knowledge of habitat preference of 135 salient wild plant species were collected (2908 records, 44 interviewees). Data collected indoors were counterchecked during outdoor interviews and participatory field work.

**Results:**

Csángós used a rich and sophisticated vocabulary to name and describe habitat categories. They distinguished altogether at least 142–148 habitat types, and named them by 242 habitat terms. We argue that the method applied and the questions asked (‘what kind of place does species X like?’) helped the often implicit knowledge of habitats to be verbalized more efficiently than usual in an interview. Habitat names were highly lexicalized and most of them were widely shared. The main features were biotic or abiotic, like land-use, dominant plant species, vegetation structure, successional stage, disturbance, soil characteristics, hydrological, and geomorphological features. Csángós often used indicator species (28, mainly herbaceous taxa) in describing habitats of species. To prevent reduction in the quantity and/or quality of hay, unnecessary disturbance of grasslands was avoided by the Csángós. This could explain the high number of habitats (35) distinguished dominantly by the type and severity of disturbance. Based on the spatial scale and topological inclusiveness of habitat categories we distinguished macro-, meso-, and microhabitats.

**Conclusions:**

Csángó habitat categories were not organized into a single hierarchy, and the partitioning was multidimensional. Multidimensional description of habitats, made the nuanced characterization of plant species’ habitats possible by providing innumerable possibilities to combine the most salient habitat features. We conclude that multidimensionality of landscape partitioning and the number of dimensions applied in a landscape seem to depend on the number of key habitat gradients in the given landscape.

## Background

Vegetation- and habitat-related knowledge of human societies has been accummulating for millennia. Vegetation and landscape knowledge that developed independently (or mostly independently in European landscapes) of science is studied by ethnobiology. Ethnobiologists seek to understand how different peoples perceive, classify, and mentally process the living world, and how they then apply that knowledge [1]. In the last decades, habitat and vegetation-related knowledge of many peoples was studied. However, it was only Johnson and Hunn [2] who introduced the term landscape ethnoecology. Landscape ethnoecology focuses on the ecological features of a landscape (e.g. ecotopes, habitats, vegetation types, and other landscape elements), and aims to understand how the living landscape is perceived, named, imagined, classified, and managed by people who live in it.

Traditional habitat knowledge has been documented in detail in only a few cases in Europe: in the French Alps [3,4], in the Hortobágy salt steppe in Hungary [5], and based on toponyms in two areas in Transylvania, Romania [6,7]. There are probably a number of reasons for this. First, this sort of traditional knowledge may have been lost by now or is at the verge of extinction in Western Europe [8]. Second, Western European ethnobiologists tend to work outside of Europe almost without exception. Meanwhile, in the countries of Eastern and Southeastern Europe, which are still rich in this sort of traditional knowledge, this type of research is missing. It is surprising that studies specifically focusing on traditional folk knowledge of vegetation have appeared in the international literature only in recent years [9-26].

In Europe, traditional land-use and the connected traditional ecological knowledge have survived mostly in agriculturally marginal areas. Our research was carried out in a mountainous environment, in the zone of spruce forests of the Carpathians. In this environment, hay meadows and highland pastures alternate with spruce forests. The species-rich grasslands of this zone are all anthropogenic in origin [27]. As they were formed after deforestation, they tend to be re-occupied by forests when regular management ceases [27,28]. Thus, their survival depends on the continuity of human intervention. The maintenance of grasslands (hay meadows, pastures) requires precise knowledge of the habitats from both traditional farmers and nature conservationists. However, traditional knowledge related to these habitats has not yet been well documented.

Traditional ecological knowledge is transferred from generation to generation, while in a traditional community each generation adds its own observations [6,29,30]. Farming based on traditional knowledge often created landscape utilization patterns that survived on the long-run [31-33]. Meilleur [3] argues that in the French Alps (Les Allues Valley), landscape pattern is regarded as essentially unaltered, and farming practices remained similar from the (14^th^) 16^th^ to the beginning of the 20^th^ century. However, Meilleur [3] had to rely on living memory in his ethnobiological study, as traditional farming had been abandoned by the 1970s-1980s. Even so, he was able to document nuanced knowledge of habitats (20 main habitat types [3,4]). These were used by the local people in a well developed farming system of fullfield agriculture, arboriculture, and forestry along with mowing, grazing, animal-husbandry, and confection of milk products. It is worth noting that farming practices in the Les Allues region in the first half of the 20^th^ century seem to be surprisingly similar to those that are still practiced in our study area in the Eastern Carpathians.

Traditional land-use in the Carpathians is also based on detailed local knowledge of vegetation and habitat. This has already been documented in two landscapes: in Kalotaszeg, west of Kolozsvár (Cluj) and in the Gyergyó Basin, in the Eastern Carpathians [6,7]. These authors elicited the living knowledge of plant species, but did not study the living knowledge of habitats. Instead, they reconstructed folk habitat categories found in the landscape by linguistic methods based on historical and contemporary toponyms. Even with this methodology they were able to reveal a very detailed habitat vocabulary.

While folk plant (and also animal) classifications are usually hierarchical see e.g. [1], Hunn and Meilleur [22] found that habitat categories tend to be ordered into a single, but less hierarchical (shallow) landscape ethnoecological partition. Phytosociological units used by botanists are also classified into a single, though highly structured hierarchy see e.g. [34]. Hunn and Meilleur [22], Fleck and Harder [35], and Shepard et al. [11] argue that folk habitat classifications are usually multidimensional. Our preliminary study [36] also showed signs of shallow hierarchy and multidimensionality. We consider landscape partitioning to be multidimensional if several distinct sets of salient environmental features are used – consciously or unconsciously – to define habitats, and thus habitats cannot be arranged along a single dimension.

In this paper, the traditional habitat knowledge of Csángó people living in Gyimes (Eastern Carpathians, Romania) is introduced. We identify the habitats into which Csángós partition their mountainous landscape and the features they use to distinguish these habitats. We also document the role of species composition in differentiating habitats, and determine whether it is possible to arrange folk habitat categories into a single landscape ethnoecological partitioning.

## Materials and methods

### The landscape

**Figure 1:**
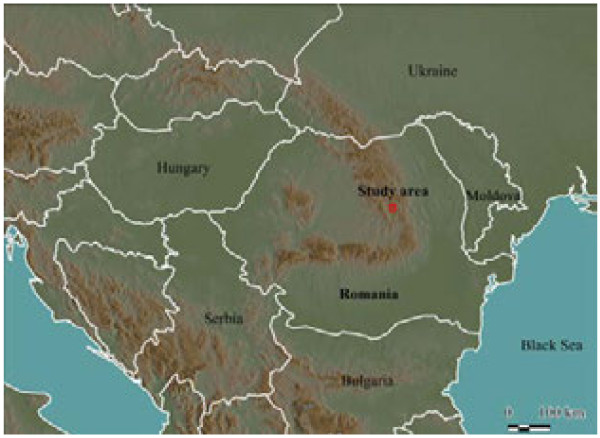
The study area - Gyimes - in the Eastern Carpathians, Romania, Central Europe.

**Figure 2:**
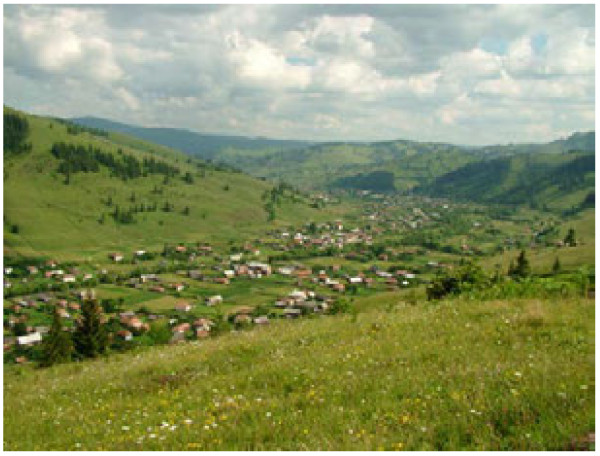
The valley bottom with the village in the study area.

**Figure 3:**
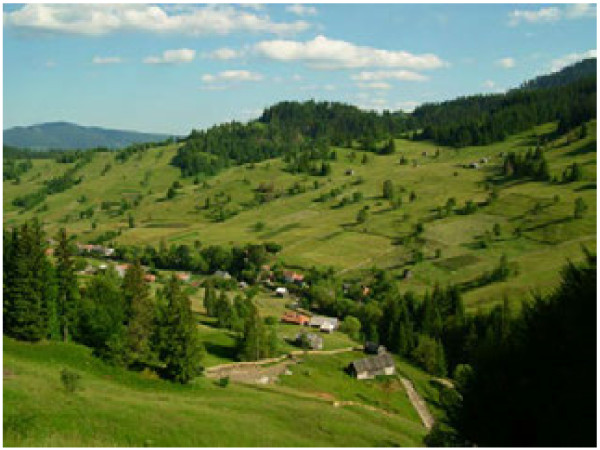
Inner hay meadows near the settlement, potato fields, houses and forests in Gyimes (Romania).

**Figure 4:**
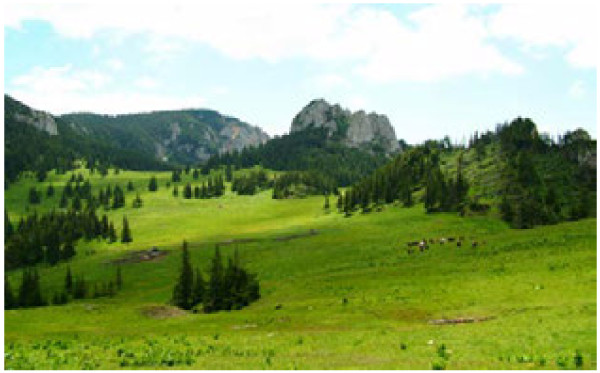
Mountain landscape with pastures, forests and rock outcrops in Gyimes (Romania).

The study area (Gyimes), is located among the mountain ridges of the Eastern Carpathians (coordinates: N 46°37’22.45”, E 25°57’24.06”) (Figures 1, 2, 3, 4). This part of the Carpathians is made up of sandstone ridges, although limestone also reaches the surface in the study area. The Eastern Carpathians are not a compact mountain range, as it is dissected by several valleys and basins; it is formed by crystalline rocks, and flysch [37]. The elevation varies between 900 and 1500 m. The highest peak is the Naskalat (1553 m). The climate is montane (boreal). The yearly mean temperature ranges from 4 to 6°C, and the annual precipitation varies from 700–800 mm in the valleys to 1000–1200 mm on the mountains [38]. The most significant river is the Tatros with several tributaries of which the largest is the Hidegség. Our study area was restricted to the extensive valley system of the latter.

**Figure 5:**
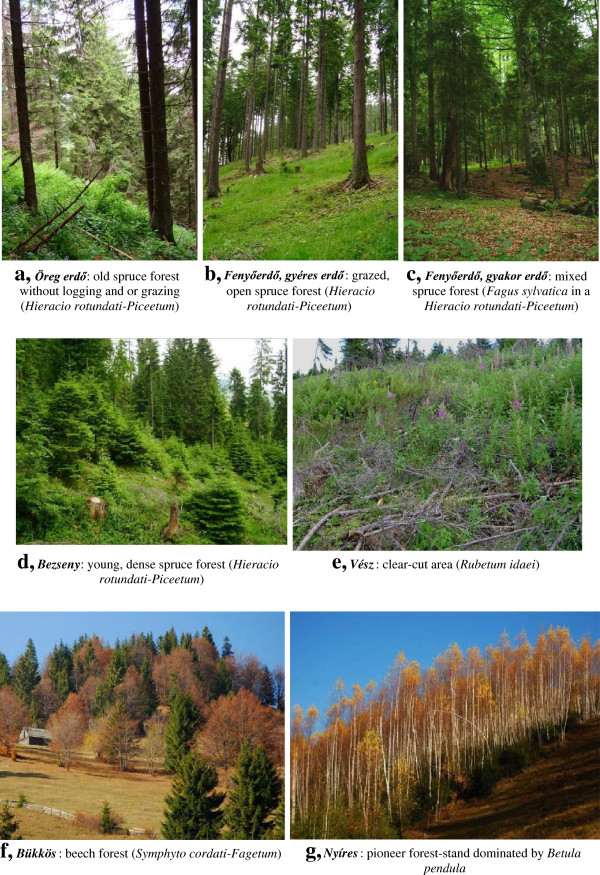
**Forest habitats in Gyimes (Eastern Carpathians). *a, Öreg erdő***: old spruce forest without logging or grazing (*Hieracio rotundati-Piceetum*). ***b, Fenyőerdő, gyéres erdő***: grazed, open spruce forest (*Hieracio rotundati-Piceetum* ). ***c, Fenyőerdő, gyakor erdő***: mixed spruce forest (*Fagus sylvatica* in a *Hieracio rotundati-Piceetum*). ***d, Bezseny***: young, dense spruce forest (*Hieracio rotundati-Piceetum*). ***e, Vész***: clear-cut area (*Rubetum idaei*). ***f, Bükkös***: beech forest (*Symphyto cordati-Fagetum*). ***g, Nyíres***: pioneer forest-stand dominated by *Betula pendula*

**Figure 6:**
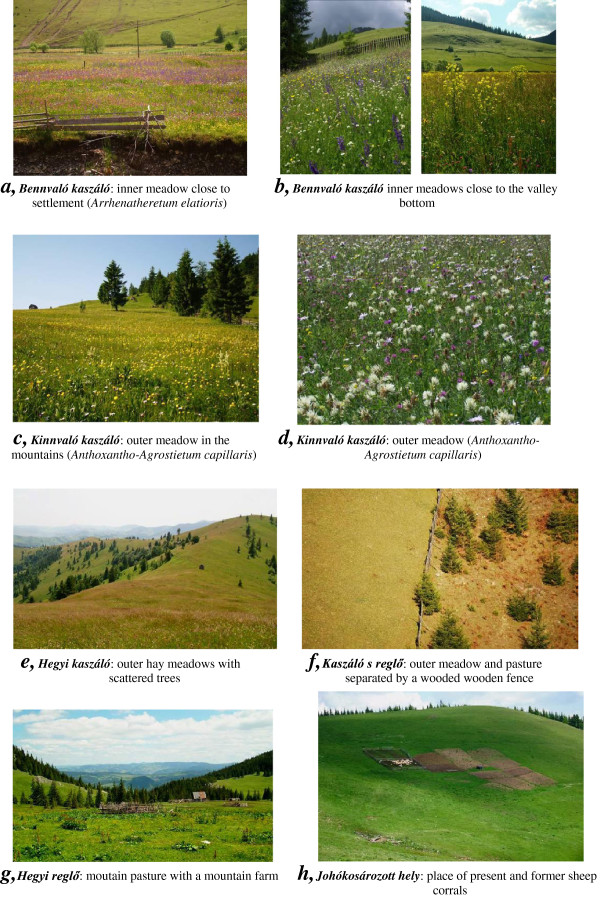
**Grassland habitats in Gyimes (Eastern Carpathians). *a, Bennvaló kaszáló***: inner meadow close to settlement (*Arrhenatheretum elatioris*). ***b, Bennvaló kaszáló*** inner meadows close to the valley bottom. ***c, Kinnvaló kaszáló***: outer meadow in the mountains (*Anthoxantho-Agrostietum capillaris*). ***d, Kinnvaló kaszáló***: outer meadow (*Anthoxantho-Agrostietum capillaris*). ***e, Hegyi kaszáló***: outer hay meadows with scattered trees. ***f, Kaszáló s reglő***: outer meadow and pasture separated by a wooden fence. ***g, Hegyi reglő***: moutain pasture with a mountain farm. ***h, Johókosározott hely***: place of present and former sheep corrals

**Figure 7:**
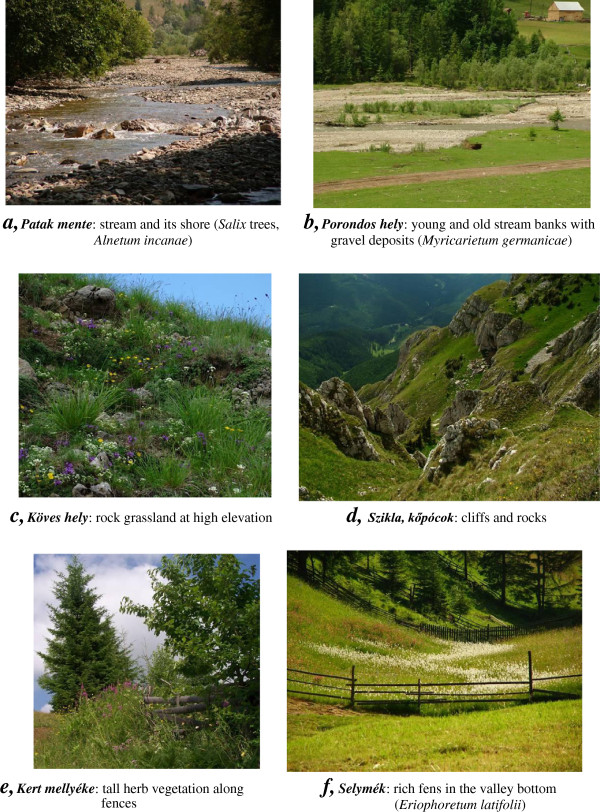
**Wet and stony habitats in Gyimes (Eastern Carpathians). *a, Patak mente***: stream and its shore (*Salix* trees, *Alnetum incanae*). ***b, Porondos hely***: young and old stream banks with gravel deposits (*Myricarietum germanicae*). ***c, Köves hely***: rock grassland at high elevation. ***d, Szikla, kőpócok***: cliffs and rocks. ***e, Kert mellyéke***: tall herb vegetation along fences. ***f, Selymék***: rich fens in the valley bottom (*Eriophoretum latifolii*)

This area falls within the Carpathian district of the Central European Floristic Region. Its vegetation is very diverse with many habitats (see Figures 5, 6, 7). The most common forest tree is spruce (*Picea abies*). The majority of the 614 vascular plant species we documented have Eurasian (29%), European (13%) and circumpolar (12%) distributions. The number of relicts of the Quaternary Period is high [39]. Typical endemic species are e.g. *Viola declinata*, *Campanula carpathica*, and *Hepatica transylvanica*.

Almost the entire area was formerly forested, and lies within the belt of spruce forests between (600) 1200 – 1600 m. Beech forests also occur, but are very limited in extent (for additional plant associations described by phytosociologists based on dominant, characteristic, and differential plant species typical of the study area, see Table 1. ^a^ .

**Table 1:** Plant associations in the Gyimes region (Eastern Carpathians)

	
*Hieracio rotundati-Piceetum* Pawl. Et Br.-Bl. 1939	(Figure 5a,b,c,d)
*Symphyto cordati-Fagetum* Vida 1959	(Figure 5f)
*Rubetum idaei* Pfeiff. 1936 em. Oberdorfer 1973	(Figure 5e)
*Calamagrostio arundinaceae-Digitalietum grandiflorae* (Sill. 1933) Oberdorfer 1957 (Syn.: *Calamagrostio-Spireetum ulmifoliae* Resm. et Csűrös 1966)	(Figure 7e)
*Senecioni sylvatici-Epilobietum angustifolii* R. Tüxen 1937	(Figure 5e)
*Fragario-Rubetum* (Pfeiffer 1936) Sissingh 1946	
*Scorzonero roseae – Festucetum nigricantis* (Puscaru et al. 1956) Coldea 1987	
*Arrhenatheretum elatioris* Br.-Bl. 1919 – *festucetosum rubrae* Tüxen 1951	(Figure 6a)
*Festuco rubrae-Agrostetum capillaris* Horv. 1905	(Figure 6c,d)
*Anthoxantho-Agrostietum capillaris* Sillinger 1933	
*Violo declinatae – Nardetum* Simon 1966	(Figure 6e,h)
*Salici purpureae-Myricarietum* Moor. 1958	(Figure 7b)
*Salicetum triandrae* Malcuit 1929	(Figure 7b)
*Aegopodio-Alnetum* V. Kárpáti, I. Kárpáti & Jurko 1961	(Figure 7b)
*Telekio speciosae-Alnetum incanae* Coldea (1986) 1990	(Figure 7a)
*Telekio-Petasitetum hybridi* (Morariu 1967) Resm. et Ratiu 1974	
*Carici flavae-Eriophoretum latifolii* Soó 1944	(Figure 7f)
*Caricetum rostratae* Rübel 1921	
*Equisetetum fluviatilis* Soó 1947	
*Glycerietum plicatae* (Kulcz 1928) Oberdorfer 1954	
*Scirpetum sylvatici* Maloch. 1935 em. Schwich 1944	
*Typhetum shuttleworthii* Soó 1927	
*Chenopodio vulvariae-Urticetum urentis* (Slavnic 1951) Soó 1971	
*Matricarietum discoideae-recutitae* Jarolímek et al. 1997	
*Poo compressae-Tussilaginetum* R. Tüxen 1931	
*Arctietum lappae* Felföldy 1942	
*Cirsio lanceolati-arvensis* Morariu 1943	
*Carduetum acanthoidis* Felföldy 1942	
*Sambucetum ebuli* Felföldy 1942	
*Aegopodio-Petasitetum hybridi* R. Tüxen 1947	
*Trifolio medii-Melampyretum nemorosi* Dierschke 1974 etc.	(Figure 7e)

### Local people in Gyimes: the Csángós

The Gyimes area was a political border zone of the Hungarian Kingdom with no record to its intensive use before the 18^th^ century [40,41]. The area was owned by the villages 14–20 km to the west on the other side of a high mountain range. Early 17^th^ century archive sources document the construction of a border protection system, to which food and other resource was provided by the above villages [40]. The souces do not mention any permanent human populations in the Gyimes area. Sources reporting on the Tatarian raids in 1694 do not refer to any settlements or inhabitants in the Gyimes either [40]. The first church was built only in 1782 close to the border [40]. The Gyimes area was covered with 75% forest in 1792. There were narrow stripes of grasslands and roads in the main valleys (Tatros). Exploitation of the timber resources in the area became possible only after the building of the railway in 1897. These data suggest that the area was settled comparatively late. Immigration from the west (Transylvania) and east (Moldva), and deforestation began only in the 18^th^ century. The forested area was rapidly reduced in order to create pastures and hay meadows [40]. About 45% of the original forests were cut in the first half of the 19^th^ century [42]. The pattern of the landscape was stabilized afterwards, and the resulting grassland-forest mosaic characterizes the landscape structure even today [40].

The Gyimes Csángó is an ethnic group with about 14,000 members living in the valleys of the Tatros and its tributaries. Their native tongue is Hungarian. The Hungarian language is an agglutinative language that belongs to the Uralic language family. The Csángós in Gyimes speak a specific dialect (a transition between the Moldavian Csángó and the Transylvanian dialects). Their culture, particularly their dance and folk song culture, folklore, religious life, dress, characteristic life-style and customs, which were all in use until recently, have preserved a number of archaic elements see e.g. [40,43-45]. The local culture has been under transformation since the change in the political system in 1989, and increasingly depends on market conditions, while emigration has started. The delayed onset of cultural transformation was due to the minority status of the Csángó community. Preservation of the spoken language and the characteristic cultural traditions was an act strengthening local identity and expressing national identity of the community.

In the second half of the 20^th^ century during communism, socialist cooperatives did not exist or only partially functioned in the Csángó territory. Csángós own their land (arable fields, pastures, meadows, and partly also the forests).

The smallest economic unit in Gyimes is the family. An average family owns 3,8 ha of land [46]. Csángós are famous for their inter-familial cooperations called *kaláka*. During periods of intensive work (e.g. hay making, construction of a house), families help each other on a reciprocal basis.

The Gyimes people still live in a comparatively semi-subsistence farming system. Animal husbandry and small-scale dairy production are the key sources of income- in their local economy [46,47]. In other alpine farming systems like the one in Gyimes, the contribution of animal husbandry to the total food output of farming was more than 80 % [48]. At present, 61% of the income in an average family comes from non-agricultural activity, 17 % from subsidies, and 22 % directly from marketing agricultural products [46].

The period suitable for grazing is limited to May-October. For this reason, a large amount of hay has to be collected and stacked. Csángós greatly depend on the quantity and quality of the biomass produced in their landscape. Until recently, a substantial proportion of the hay was mown by hand, but then the scythe was replaced by small motorized mowers. Owing to agri-environmental subsidies, mowing with scythe is again slightly spreading. Arable agriculture has lower importance. The only crop Csángós produce in large quantities is potato, the surplus of which is sold at the market or exchanged for grapes, or vegetables. The role of gathering to supplement their diet is considerable (mushrooms, *Rubus idaeus*, *Vaccinium* spp., *Fragaria* spp.). According to our own estimates, Csángós spend approximately 210 days yearly outdoors [49]. Csángós possess significant ecological knowledge that is utilized in their traditional farming activities [36,42,49]. They know many of the plant (and also animal) species in their environment. They recognize at least 294 plant species, which they classify into 207 folk taxa [42].

### Data collection and analysis

We conducted our study in the community of Gyimesközéplok (Lunca de Jos), in Hidegségpataka (Valea Rece) during 202 field days between 2005 and 2011. Data on the knowledge of plants and habitats, and the knowledge related to vegetation dynamics were collected from 44 people, of which 4 were younger than 20 years, 11 were between 20 and 60 years, and 29 were older than 60 years. All live in Gyimesközéplok-Hidegségpataka, and most of them were also born there. A small fraction was born in Háromkút (Trei Fântâni) founded by Csángós from Gyimesközéplok. Data were collected during indoor and partly outdoor semi-structured and structured interviews, and participatory observations [50].

First, local names of wild plants were collected. Most plant species have only a single local name, and the number of synonyms is low [49]. This suggests that the knowledge of plant species is still actively used, and is shared widely in the community. We asked questions about the habitat preferences of 135 of the identified plant folk taxa. To avoid confusion, we identified uncertain species with the help of colored paintings [51]. After testing different questions, we collected data by asking the question of ‘what kind of place does species X like?’. The question of ‘where does species X grow’ brought answers on the specific locality the species occurred in. With additional questions (e.g. ‘what should we know of this species/habitat?’) we gained detailed ecological characterization of the habitats. The reliability of our data was checked during participatory observations in the field. We asked people to define and describe the ambiguous habitat terms. In the field, we listened to their conversations, and also asked the name of certain spots to gain insights how Csángós structured their local environment. We collected data on the habitat requirements of 129 species / informant on average. We asked 3620 questions altogether, of which 80.3% were sufficiently detailed for further analysis (2908 records). 35 h of sound recording were transcribed. All habitat names and expressions describing a habitat were sorted out. As supplementary data collection, we collected all the geographical names from the studies of Ilyés (unpubl.) and Rab [7] that were related to habitats. With the help of three knowledgeable Csángó respondents, we selected from these the names that are still in use in Gyimes as a habitat name.

There is diverse terminology for landscape elements in ethnobiology: e.g. ecotope, habitat, kind of place, biotope [23]. We chose to use the term *habitat*, since in Europe this is the most widespread term that includes all living creatures on a piece of land with its soil, bedrock, and hydrology. A habitat is mostly defined by its vegetation, and is more or less a synonym of ecotope (the suggested term by [2,22]).

Clustering of habitat terms into sets in tables (see below) resulted more from an etic assessment on our part than from any concerted effort we undertook to explore whether they were kinds of natural sets for Csángós. We regarded all terms in the habitat sets as a habitat term irrespective of their other meanings in the local language (e.g. a type of bedrock (clay), or a type of property (garden) cf. [2]), because all originated from answers to our key question (or participatory field work or from counterchecked toponyms).

## Results

**Figure 8:**
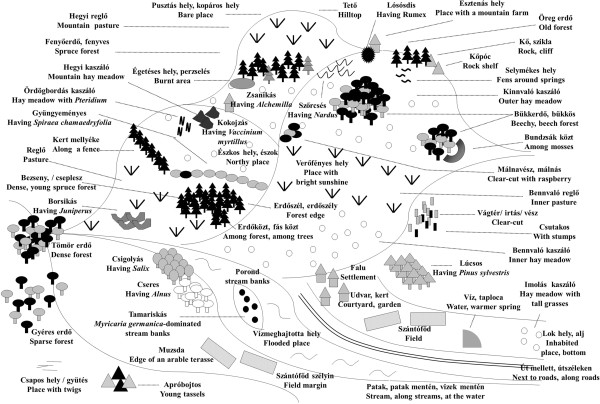
**The 49 most salient Csángó habitat categories (Gyimes, Eastern Carpathians, Romania).** For detailed English equivalents of names see Tables 2, 3, 4, 5, 6, 7, 8, 9, 10

Csángós identified altogether at least 142–148 different habitat categories, and named them by 242 terms (Figure 8). Most names were widely shared among the Csángós. Csángós identified habitats using various features: land-use, dominant plant species, vegetation structure, successional stage, disturbance, soil characteristics, hydrological, and geomorphological features.

Csángós use practically all parts of the landscape in some way or another. Thus, essentially all parts of their environment (about 99%) may be associated with a habitat type classified based on how a piece of land is used. This is the most frequent way of grouping habitats used by the local people (Table 2).

**Table 2:** Land-use related set of Csángó habitat terms, their meanings and English equivalents (literal translations of names are given in parentheses)

**Csángó habitat names**	**Meanings and English equivalents**
***kaszáló***^***1,2***^	Hay meadow (the same)
***bennvaló kaszáló***^***2***^	Hay meadow close to the settlement, fertilized every 2–3 years, mown twice a year, dominated by monocotyledons (inner hay meadow)
***kinnvaló (hegyi) kaszáló***^***2***^	Hay meadow in the mountains, further from settlements, not fertilized, mown once a year, dicotyledons are common (outer hay meadow / hay meadow in the mountains)
***erdőközötti, erdei kaszáló***^***2***^	Meadow among forests (hay meadow among forests / woodland meadow)
***reglő / nyáraló***^***1,2***^	Pasture used in summer (pasture / to spend the summer)
***bennvaló reglő***^***2***^	Pasture, close to the settlement (inner pasture)
***hegyi reglő***^***2***^	Mountain pasture (the same)
***őszlő***^***2***^	Meadow where the regrowth is grazed in the autumn (to spend the autumn)
***sarjús hely***^***2***^	A mown place with regrowth (place with regrowth)
***kert / udvar***^***2***^	Inner meadow among the houses, fertilized every 2–3 years, and mown 2–3 in a year (garden / courtyard)
***kert mellett, kertszély***^***2***^	Bushy or tall-herb or weedy vegetation along fences (along a fence / edge of a garden)
***szántófőd, pityókafőd, gabonafőd***^***2***^	Field, potato field, cereal field (the same)
***szántófőd szélin***^***3***^	Field margin (the same)
***felhagyott kaszáló***^***2 ***^***/ felhagyott szántóföld***^***2***^	Abandoned hay meadow, abandoned arable field (the same)
***erdő***^***1,2***^	Forest (the same)
***vad hely***^***1,2***^	Area where vegetation is not controlled by humans (usually an old forest in narrow valleys) (wild place)

Superscripts indicate scale: ^1^macrohabitat; ^2^mesohabitat; ^3^microhabitat.

These habitats (20 habitats, e.g. meadow, pasture, arable field, woodland, settlement, Figures 5, 6) cover the largest geographical area, and formed the basis of Csángó landscape ethnoecological partitioning. For defining habitats of plant species, Csángó people used these land-use habitat terms, completing them with additional, more refined categories.

The presence of a dominant plant species is often a salient feature in the more detailed partitioning of the landscape (31 habitats, Table 3). These categories were not defined by their species composition (as scientific plant associations are). Instead, these terms indicated only the dominant or most salient species of the area described. Besides dominant species (such as *Picea abies,* Figure 5b,c, *Nardus stricta,* Figure 6h), locally abundant, ecologically conspicuous species were used for the recognition and naming of habitats (for example, *Betula pendula*, Figure 5g, *Acer pseudoplatanus, Sorbus aucuparia, Taxus baccata, Vaccinium myrtillus*).

**Table 3:** Vegetation related set of Csángó habitat terms, their meanings and English equivalents (literal translations of names are given in parentheses)

**Csángó habitat names**	**Meanings and English equivalents**
***fenyőerdő, fenyves, fenyőfás***^***2***^	Coniferous forest, dominated by *Picea abies* and *Abies alba,* rarely mixed with *Fagus sylvatica* (spruce forest, with spruce, with spruce trees)
***bükkerdő, bükkös / leveles erdő***^***2***^	Deciduous forest, dominated by *Fagus sylvatica* mostly mixed with *Picea abies* (beechy, beech forest / leafy forest).
***lúcsos***^***2***^	Planted forest of *Pinus sylvestris* often with *Juniperus communis* (having *Pinus sylvestris*)
***cseres (cserfás) / csigolyás / ficfás, füzes***^***2***^	Forest stands along streams, dominated by *Alnus incana* or bushy *Salix* spp. or *Salix* spp*.* trees (having alder / having bushy willow / having *Salix*)
***nyírfás / rakottyás / nyárfás***^***2***^	Pioneer forest stands dominated by *Betula pendula* or *Salix caprea* or *Populus tremula* (having birch / having *Salix caprea* / having aspen)
***jáhoros / kórusos / tiszás***^***2***^	Deciduous forest stands, in which *Acer pseudoplatanus*, *Sorbus aucuparia* or *Taxus baccata* are characteristic (having maple / having *Sorbus* / having *Taxus*)
***gyüngyeményes / fügés / bojzás***^***2***^	Scrub habitat, dominated by *Spiraea chamaedryfolia* or *Ribes uva-crispa* or *Sambucus racemosa*, *S. nigra* (having *Spiraea /* having *Ribes* / having *Sambucus*)
***sátés, sásos***^***2***^	Rich fens and swamps, dominated by *Carex* spp. (having sedges)
***nádas***^***3***^	A marshy area, dominated by *Typha angustifolia* or *T. shuttleworthii* (having reed)
***békalábas, surlós***^***2***^	Wetlands, dominated by *Equisetum palustre* (having *Equisetum palustre*)
***podbállapis***^***3 ***^***/ keptelános***^***2***^	A pioneer surface dominated by *Tussilago farfara* or an area with *Petasites*-dominated tall-herb vegetation along streams (having *Tussilago* / having *Petasites*)
***kokojzás***^***2 ***^***/ ménisorás***^***2 ***^***/ takonykokojzás***^***2***^	*Nardus* grasslands that are characterized by *Vaccinium myrtillus*, *Vaccinium vitis-idaea* or *Vaccinium gaultherioides* (having blueberries / having cowberries / having bogberries)
***szőrcsés***^***2***^	Grasslands, mainly pastures, dominated by *Nardus stricta* (having *Nardus*)
***danciás***^***2***^	Grasslands, where *Gentiana lutea* grows (having *Gentiana*)
***imolás***^***2***^	Grasslands, dominated by tall grasses mainly *Trisetum flavescens* (lit hay meadow with tall grasses)
***zsanikás***^***2***^	Grasslands, mainly pastures, dominated by *Alchemilla* species (having *Alchemilla*)
***ászpás***^***2***^	Mountain hay meadows and pastures with *Veratrum album* stands (having *Veratrum*)
***borsikás***^***2 ***^***/ hecsellis***^***2***^	Pioneer stands on pastures, dominated by *Juniperus communis* or *Rosa canina* agg. (having *Juniperus* / having *Rosa*)
***csipkés***^***2***^	Degraded stands on pastures and fields, dominated by *Cirsium* and *Carduus* spp. (having thistles)
***csihányos***^***2 ***^***/ lósósdis***^***2***^	Degraded, nutrient rich stands dominated by *Urtica dioica* or *Rumex alpinus* (having nettles / having *Rumex*)

Superscripts indicate scale: ^1^macrohabitat; ^2^mesohabitat; ^3^microhabitat.

Vegetation structure also was a salient feature (15 habitats, Table 4, Figure 5b,c). Csángós primarily separated wooded and treeless habitats on the basis of vegetation structure. In the case of forests, the structure of the tree stands and the characteristics of the forest edges were salient features.

**Table 4:** Vegetation structure related set of Csángó habitat terms, their meanings and English equivalents (literal translations of names are given in parentheses)

**Csángó habitat names**	**Meanings and English equivalents**
***erdőközt, fás közt***^***2***^	In the forest (among forest / among trees)
***erdőszél, erdőszély***^***2***^	Forest edge (the same)
***fa mellett, fa alja, fa töve***^***3***^	Under a tree, next to a tree trunk (at (under) tree)
***gyéres erdő***^***1,2***^	Thinly grown or partly cleared forest (sparse forest)
***tömör (gyakor) erdő***^***1,2***^	Dense forest (dense, frequent forest)
***erdőközötti puszta***^***3 ***^***/ lik***^***3***^	Clearings in the forest or a smaller opening in a forest (open place between forests / hole)
***csoporterdő, erdőcsoport***^***2***^	Fragmented forest stands (forest group)
***málnaveszes szélye***^***2***^	Edge of a clear cut (edge of a raspberry dangerous place)
***bokros hely, bokrok közt, bozót***^***2***^	Bushy area, often diverse, mainly also small trees (bushy / thicket)
***árnyékos hely***^***2,3***^	Shaded area (the same)
***rét, mező, nyílt terület***^***1***^	Grassland in an open, relatively flat landscape (meadow / grass / open area)
***pusztás hely, kopáros hely***^***2***^	Mountain top without forests, often not inhabited or large opening in a forest (bare place)
***gyepes hely, füves, fű közt, pázsint, pástos hely***^***2***^	Area covered with grasses, often on a layer of gravel (grassy place, lawn)
***bundzsák közt***^***3***^	Among mosses (the same)
***kaszáló szély***^***2***^	Edge of a mountain hay meadow (the same)

Superscripts indicate scale: ^1^macrohabitat; ^2^mesohabitat; ^3^microhabitat.

Csángós also recognized and named habitats created by natural or human disturbances. The most significant of these disturbances was clear cutting. The successional stages following clear cutting were all clearly separated in detail (14 habitats, Table 5, Figures 5e, 6h, 7b). In some cases, they even distinguished two types of clear-cuts: places of former spruce and beech forests. In the case of abandoned arable fields, Csángós did not distinguish additional stages besides the one dominated by *Taraxacum officinale*, which was considered as the first colonizer. Older grassy fields developed over abandoned fields were called hay meadows and were not distinguished in any other way.

**Table 5:** Successional stage related set of Csángó habitat terms, their meanings and English equivalents (literal translations of names are given in parentheses)

**Csángó habitat names**	**Meanings and English equivalents**
***vágtér / irtás / vész***^***2***^	Cleared area, often turned into a grassland usually with twigs all over (cut-area / clearing / dangerous)
***csapos hely / gyütés***^***3***^	Area where twigs are humped on a clear cut (place with twigs / collection)
***csutakos***^***2***^	A cleared area with stumps (with stumps)
***epervész, epres vágtér***^***2***^	An area with *Fragaria* spp. on clear cuts (dangerous with *Fragaria*, *Fragaria* cut area)
***málnavész, málnás***^***2***^	An area with *Rubus idaeus* on clear cuts (dangerous with raspberries, having raspberries)
***apróbojtos***^***2***^	Young spruce forest, height less than 1 m (young tassels)
***bezseny, cseplesz***^***2***^	Dense, 5–10 years old young spruce forest (specific local expressions reflecting on density and small tree size)
***bezsenyes erdő***^***2***^	Transitional stage between young tassels and young spruce forest (?)
***fiatal erdő***^***2***^	Young spruce forest, ca. 10 years old (young forest)
***karós erdő***^***2***^	Forest with stake sized trees, dbh 7–20 cm (staked forest)
***boronaerdő***^***2***^	Forest with trees good for house building, dbh 25–30 cm (beam forest)
***szelhás erdő (szelhaerdő)***^***2***^	Forest with straight (and older) trees, the trees are good for shingle, dbh min. 20 cm, age of the forest is at least 40 years (?)
***kinőtt erdő / öreg erdő / nagy erdő***^***1,2***^	Old forest, above 70–100 years (adult forest / old forest / large forest)

Superscripts indicate scale: ^1^macrohabitat; ^2^mesohabitat; ^3^microhabitat.

Additional disturbances creating places were manuring, trampling and grazing of animals, and the presence of once settled sites (15 habitats, Table 6). In addition to their biotic and abiotic features, these places were often characterized by indicator species (i.e. *Rumex alpinus,* Figure 6g, *Urtica dioica*, *Potentilla anserina*). Habitats that are named after natural disturbances were gravel deposits in and adjacent to streams (Figure 7b), and rarely windfall or burnt areas in forests. Habitats in the neighborhood of buildings and roads were particularly disturbed. Habitats in this set were characteristically weedy in species composition (6 habitats, Table 7).

**Table 6:** Disturbance related set of Csángó habitat terms, their meanings and English equivalents (literal translations of names are given in parentheses)

**Csángó habitat names**	**Meanings and English equivalents**
***ganyés, trágyázott hely***^***2***^	Manured site, a nutrient rich area (manured place)
***johókosározott hely***^***2,3***^	Place of former sheep corrals (the same)
***esztenás hely***^***3***^	A nutrient rich area around mountain farms, or on the place of sheep corrals (place with a mountain farm)
***ahol az állatok kitapossák***^***3***^***, tapodott hely***^***2 ***^***/nem tapodott hely***^***2***^	Areas trampled (or not) by animals or humans (trampled by animals/ trampled area / not trampled area)
***ahol legelnek a marhák***^***2***^	Synonym of pasture (where cattle graze)
***hangyaboly***^***3***^	Anthill on the meadows and pastures, the main habitat of *Thymus*-species (anthill)
***gyomos hely***^***2***^	A degraded place dominated by weeds (weedy place)
***felmaradt épület-hely***^***3***^	Area of a former mountain building (abandoned building place)
***friss vágtér***^***2***^	New clear cut area (the same)
***vízmeghajtotta helyeken***^***2***^	Flooded area with erosion and accummulation of gravel and sand (flooded place)
***égetéses hely, perzselés***^***2,3***^	A burnt area, usually recovered by forest or a singed area, usually *Nardus* or *Juniperus* was singed (burning / singeing)
***aszalás***^***3***^	An area where spruce trees were ring-girdled (desiccated)
***suvadás, suvadós hely***^***2,3***^	An area with characteristic landslide (slide, slidey place)
***martos, mart***^***3***^	Suddenly steep part of an area (bitten)
***mocskos hely***^***3***^	Small organic garbage dump or pile of twigs, e.g. along fences, on stream banks (dirty place)

Superscripts indicate scale: ^1^macrohabitat; ^2^mesohabitat; ^3^microhabitat.

**Table 7:** Habitat set of Csángó habitat terms applied to highly disturbed areas, their meanings and English equivalents (literal translations of names are given in parentheses)

**Csángó habitat names**	**Meanings and English equivalents**
***épületek mellett, házak szélén***^***3***^	Close to buildings and houses (the same)
***istállók körül, mellett***^***3***^	Around the barn, close to the barn (the same)
***út mellett, útszéleken***^***2,3***^	On road edges (next to roads / along roads)
***ösvenyek mentin***^***2,3***^	Along paths, a trampled habitat (along paths)
***kert mellyéke***^***2***^	Along a fence (the same)
***sánc, árok***^***3***^	Ditch, along ditches (ditch)

Superscripts indicate scale: ^1^macrohabitat; ^2^mesohabitat; ^3^microhabitat.

Csángós identified many habitats by abiotic features (e.g. soil, hydrology, geomorphology). Many names contained the word ‘place’ (e.g. thin place, stony place, wet place). Part of the descriptive characteristics based on soil types was supplemented by geological details (bedrock type) (15 habitats, Table 8, Figure 7c,d). The partitioning of wetlands also was detailed (12 habitats, Table 9, Figure 7a,f). Given that the area is mountainous, topographic and geomorphological features were salient (17 habitats, Table 10). For some habitats, elevation (a.s.l.) and exposure were the most salient features.

**Table 8:** Soil type and bed rock related set of Csángó habitat terms, their meanings and English equivalents (literal translations of names are given in parentheses)

**Csángó habitat names**	**Meanings and English equivalents**
***kő, szikla***^***2,3***^	Boulder, rock (the same)
***kőpóc***^***3***^	Small grassland patches on flat rock surfaces on rock cliffs (rock shelf)
***köves hely***^***2,3***^	Rocky area with open vegetation (stony place)
***palaköves***^***2***^	Place with slates (the same)
***fehér kő, mészkő***^***2,3***^	Limestone (white rock, limestone)
***kőcsúszásos hely***^***2***^	Scree (place where rocks slide)
***kavicsos hely***^***2***^	Area with gravel (gravelly place)
***agyagos hely***^***2***^	Area with loamy soils (loamy place)
***nyirkos hely***^***3***^	A damp area often under trees (damp place)
***iszapos hely***^***2***^	Area with muddy soils (muddy place)
***homokos hely***^***2***^	Sandy soils (sandy place)
***lágy / nedves hely***^***3***^	‘Soils’ of wetlands (soft / damp place)
***erős, szikár, szikonyos***^***1,2***^	Area with dry soils (strong place / barren / ?)
***kövér, zsíros hely***^***1,2***^	Nutrient rich area (fat place)
***sovány, silány hely***^***1,2***^	Nutrient poor area (thin place)

Superscripts indicate scale: ^1^macrohabitat; ^2^mesohabitat; ^3^microhabitat.

**Table 9:** Hydrology related set of Csángó habitat terms, their meanings and English equivalents (literal translations of names are given in parentheses)

**Csángó habitat names**	**Meanings and English equivalents**
***selymékes, selyke, sepedékes hely, tepsányos***^***2***^	Fens around springs, mainly sedge-dominated stands, rarely with *Sphagnum* (a sinking area)
***mocsaras hely***^***2***^	Muddy areas around springs or along streams, with sedge-dominated stands (marshy place)
***sátészély***^***2,3***^	Edge of a fen (the same)
***félvizenyes hely, szinte olyan vizenyes, s mégse***^***3***^	Partly waterlogged soils on the margin of fens (half-watered place)
***vizes hely, vizenyes terület***^***1,2***^	Wetlands (wet place)
***víz, taploca***^***2***^	Warmer spring and its creek, that never freezes (water, ?)
***vízszélye***^***2***^	At the edge of waters (the same)
***lapos, ahol a víz elterül***^***2***^	Flooded place (flooded, where it is flooded)
***forrásfej, kicsi források mellett***^***3***^	Spring (spring head / around small springs)
***patak, patak mentén, patak szélén, vizek mentén***^***1,2***^	Smaller stream or along streams (stream, along streams, at the water)
***tócsa, pocsolya***^***3***^	Puddle (the same)
***tó, állóvíz***^***2,3***^	Lakes (lake, standing water)

Superscripts indicate scale: ^1^macrohabitat; ^2^mesohabitat; ^3^microhabitat.

**Table 10:** Elevation, aspect, and geomorphology related set of Csángó habitat terms, their meanings and English equivalents (literal translations of names are given in parentheses)

**Csángó habitat names**	**Meanings and English equivalents**
***bennvaló hely, bent***^***1***^	Grasslands and fields in the valley among the houses (inner place)
***lokhely / alj / alvidék / falu***^***1***^	Inhabited areas in the valleys at lower elevation (inhabited place / bottom / in low region / village)
***ódal, ódalas hely***^***1***^	Side/slope of a valley (sided, sided place)
***kinnvaló hely***^***1***^	High mountain grasslands and forests (outer place)
***hegy, havas***^***1***^	High mountain grasslands and forests (mountain / snowy)
***csúf hely***^***2,3***^	Area not mown or grazed, stony or with twigs, or steep, difficult to walk through (ugly place)
***porond***^***2***^	Young and old stream banks (deposits) with gravel (elevated)
***domb, dombos hely***^***1,2***^	An area with remnant soil hummocks of fallen spruce trees (hill, hilly place)
***verőfényes hely***^***1***^	Southern slope (place with bright sunshine)
***észkos hely, észok***^***1***^	Northern slope (northy place)
***vőgy, szurduk, hajlás***^***1***^	Valley, canyon, incline (the same)
***muzsda***^***3***^	Edge of an arable terrace
***hegyi gödrök***^***3***^	Large depressions in the mountains with grasslands and surrounded by forests (mountain holes)
***tető, legmagosabb hely***^***1***^	Hilltop, highest place (the same)
***meredek***^***1***^	Steep slopes in the mountains (steep)

Superscripts indicate scale: ^1^macrohabitat; ^2^mesohabitat; ^3^microhabitat.

Csángós often applied more than one feature to describe the habitat of a plant species. We present examples of the typical habitat descriptions of two folk taxa (Table 11). We selected species whose habitat preference was uniformly assessed among the Csángós.

**Table 11:** **Examples of habitat descriptions of *Onobrychis viciifolia *and *Rosa canina *agg**

	
*Onobrychis viciifolia* (local name: *bartacin*)	*"It only likes sunny places, where it is stony."*
	*"Bartacin grows on inner mountain slopes, which are not manured. If manured, it disappears. In hay meadows."*
	*"Bartacin likes sunny slidey places. Likes stony places, and gravel bars. If flooded, with gravel, poor, bartacin grows. There is a lot here on sunny slopes in the hay meadows."*
	*“In hay meadows, on sunny places. On poor places, which are stony.”*
*Rosa canina* agg. (local name: *hecselli*)	*"On poor places, which are stony, on loamy soil. In our pasture there is a lot, where the soil is loamy, stony.”*
	*"Everywhere. On slopes, everywhere. On hay meadows, on pastures, along roads. You do not have to plant it, it spreads.”*
	*"Hecselli grows on sunny, slumping slopes, not on northern slopes. It grows everywhere here on the sunny slopes on the pasture, where it is a bit slumping, stony."*
	*"On sunny places, along roads, on pastures and hay meadows.”*

For precise habitat description of particular plant species, Csángós used indicator species. In these cases, the typical answer was: “It likes (grows in) places where species Y grows.” Twenty-eight such species were documented (*Alchemilla* spp.*, Carex* spp.*, Carlina acaulis, Fragaria viridis, Gentiana asclepiadea, Juniperus communis, Leontopodium alpinum, Lonicera xylosteum, Matricaria discoidea, Myricaria germanica, Nardus stricta, Onobrychis viciifolia, Petasites albus, P. hybridus, Picea abies, Plantago lanceolata, P. media, Ribes alpinum, Rubus idaeus, Salix caprea, Salix* spp. *(S. elaeagnos, S. pentandra, S. purpurea, S. triandra), Spiraea chamaedryfolia, Tragopogon orientalis, Tussilago farfara, Urtica dioica, Vaccinium myrtillus, V. gaultherioides, V. vitis-idaea*). Species used as indicators of other species’ habitats occurred in 3.5% of all answers. Additional indicator species were used to make a distinction between meadows based on the quality of hay (Table 12).

**Table 12:** Mountain hay meadow types, named by indicator species

**Csángó habitat names**	**Botanical meaning, and English equivalents**
***imolás kaszáló***	Manured hay meadow with the dominance of *Trisetum flavescens* and other tall grasses (hay meadow with tall grasses)
***zableveles kaszáló***	An area with grasses like *Brachypodium pinnatum*, *Dactylis glomerata*, and *Festuca pratensis* (having oat-leaved species)
***vadlóherés kaszáló***	Hay meadow at higher elevation with wild *Trifolium* spp. (hay meadow with clover)
***báránylábas-bakcekás kaszáló***	Hay meadow with a dominance of *Salvia pratensis* and *Tragopogon orientale* at low elevation (hay meadow with *Salvia* and *Tragopogon*)
***bartacines kaszáló***	An area with oversown *Onobrychis viciifolia* (hay meadow with *Onobrychis*)
***kecskekapros kaszáló***	Hay meadow with *Laserpitium latifolium*, a species difficult to mow (hay meadow with *Laserpitium*)
***zsanikás kaszáló***	Hay meadow with *Alchemilla* spp. at higher elevation (hay meadow with *Alchemilla*)
***ördögbordás kaszáló***	Hay meadow, degraded by *Pteridium aquilinum* (hay meadow with *Pteridium*)

The spatial scale of landscape partitioning was highly variable. We distinguish three groups of habitats depending on their spatial scale. Habitat categories that refer to larger areas comprising a mosaic of habitats represent macrohabitats (e.g. up in the mountains, inhabited place). Others (called mesohabitats) refer to an area with a more or less homogenous vegetation (often corresponding to scientific plant associations) (e.g. spruce forest, area dominated by *Juniperus*, clear-cut). The third group of habitats (microhabitats) indicate small areas which stand out from their surroundings or provide a special small scale environmental niche (e.g. ant hill, next to a tree trunk, at the wall of a barn). The scale of habitats is indicated in Tables 2, 3, 4, 5, 6, 7, 8, 9, 10 by upper indices.

## Discussion

### Folk habitat sets

Csángós recognized and named at least 142–148 habitats. This number is higher than in any other landscape partitioning ever studied in the world cf. [22]. Though Fleck and Harder [35] estimated 178 rainforest habitat types (104 primary and 74 secondary forest habitats) that the Matses in the Peruvian Amazon might distinguish, only 47 of them were named. Csángó habitat categories closely reflected the diverse habitat pattern of the valley bottoms. In the mountains, however, partitioning was less detailed. Consequently, the majority of folk habitats was concentrated in and around settlements, similar to what was found in the Alps [3,4].

Our high number of folk habitat categories was not simply the result of the diverse mountainous landscape, nor did it reflect solely the deep ecological knowledge of the local Csángó people, but was also the consequence of our in-depth investigation cf. [20]. We argue that the method of our research and the questions we asked made it possible to elicit such a high number of habitat terms. The knowledge of habitat preference of plant species was mostly the result of personal experience, and, as such, was mainly implicit cf. [13,26,52]. By asking the question „what kind of place does species X like?” we “forced” people to verbalize their knowledge related to habitats. Admittedly, this method prompted informants to answer a strange, culturally inappropriate question cf. [52]. We argue, however, that in this way, the rarely shared, rather implicit knowledge was verbalized more often than usual in a conversation or an interview. Even with this method there were habitat names (e.g. *tepsányos* – a type of fen) that were only elicited after many months of field work, although, as turned out later, they were known with the same meaning by all successive informants we asked.

Csángó habitat categories were more or less discrete units, but often had diffuse boundaries with neighboring types, because the selection and delimitation of prototypic types along continuous topological and topographical gradients is not obvious [4,22,24], see e.g. “*choeni ovogeshi*” – transitional zone from floodplain to uplands, documented by Shepard et al. [11]. This phenomenon is not unknown in folk biological (species) classification systems either, though classification difficulties are usually smaller in them [22], and the spatial delimitation of tokens is usually not a problem. However, classification of taxa further from the so-called core taxon at the periphery of the prototype faces similar classification difficulties (cf. prototypicality effect [53], prototype extension model [1]).

The great majority of the habitat names was known by all Csángós, which was thus part of their shared knowledge. The *kaláka* (a type of inter-familial collaboration) could be one of the key platforms of knowledge sharing. The Csángó landscape ethnoecological partitioning was highly lexicalized and had only few synonyms (all are listed in Tables 2, 3, 4, 5, 6, 7, 8, 9, 10). This is in contrast to the findings of Molnár [5,54] who reported a large number of synonyms (up to 17) in a steppe landscape, which he attributed to the erosion of knowledge, limited sharing and the diverse origin of the local community. Similarly high level of lexicalization was observed among South American tribes [10,11,35], whereas the opposite was found in Southeast Asia [20]. The reason for the latter was likely the implicit nature of knowledge and not the lack of substantive knowledge.

### Features used to distinguish habitats

Csángós distinguished habitats by the following features: land-use, dominant plant species, vegetation structure, successional stage, natural and anthropogenic disturbances, soil types, hydrological features, and geomorphology. In a similar high mountain environment in the Alps, Netting [55] and Meilleur [3,4] found similar features used in the recognition and naming of habitats. Johnson also documented the importance of similar features (physiography, hydrologic features, vegetation and wildlife habitats) among Kaska Dena, Gitksan, and Witsuwit’en First Nations in Western Canada [13,26]. The use of abiotic and biotic features (typically vegetation-related ones, e.g. physiognomy, dominant and/or salient plant species cf. [10,11]) was typical among the Csángós, as was found in a number of communities in the world e.g. [10,11,25,35]. As opposed to some tropical peoples [11,21], and the First Nations in Canada [26], Csángós usually did not consider the presence and absence of animals indicative of habitat types (but see one exception below).

Expressed distinction of primary (non human-transformed) habitats and semi-natural habitats was not typical in Csángó landscape partitioning. This is in contrast to the well-documented basic dichotomy found in the classification systems of several tropical people (see e.g. the primary forest vs. secondary forest in swidden systems) [10,11,21]. It is perhaps because the entire area has been exposed to farming or forestry activities, resulting that the whole landscape has been rather significantly altered. There was only a single – rare and small in extent – habitat type known that was characterized by the absence of anthropogenic influences: it was the ***vadas hely*** (wild place – area not affected by humans; territory of wild animals).

Csángós do their best to minimize disturbance during farming. Natural and anthropogenic disturbances usually reduce the amount and/or quality of biomass, which Csángós try to avoid. This might be a reason why disturbance was a salient feature in their landscape ethnoecological partitioning. Disturbance may have been regarded as the main feature in 29 habitats. To counteract the effect of disturbance, Csángós tended to facilitate regeneration in several ways. They, for example, sowed seeds of *Onobrychis viciifolia* cf. [55] in the dry, south-facing slopes to restore the continuity of the grass mat. Certain gaps in the grass were restored by sowing hayseeds collected in barns cf. [3], Babai and Molnár unpubl.

The overwhelming majority of ethnic groups of which landscape partitioning has been studied so far live either in the tropics or the boreal zone. As a consequence, their habitat vocabulary focuses on the dichotomy of forested and cleared, cultivated areas [10,11,15,26], but see [21,25]. In Gyimes, where 99% of the landscape is under ‘cultivation’ (arable, pasture, meadow, or managed forest), habitat categories identified by their land-use formed the basic-level habitat partition. The economic importance of grasslands is greater than that of forests and arable fields due to the greater share of animal husbandry in the Csángós’ farming economy. Hay meadows are the most significant habitats in terms of survival, because they provide fodder for winter that should last up until May. The Csángós’ management of hay meadows is meticulously detailed. Thus, the habitat set of hay meadows also was much more detailed than that of other sets (e.g. pastures, forests). Csángós distinguished altogether 48 meadow types based on their soil, exposure and dominant species. On the other hand, their partitioning of pastures was coarser than that of the herdsmen in the Hortobágy and the Fulani in Burkina Faso [5,25].

Some Csángó habitat terms are locative, especially in the geomorphological, hydrological sets (e.g. among trees, along streams), and are literally the same as those used in Amazonia by the Matsigenka [11], Maijuna [21], or by the Gitksan and Witsuwit’en in British Columbia [13,26]. Similar locative expressions were also found by Molnár in the Hortobágy steppe [54]. In many Csángó habitat terms used for abiotically salient habitats, the term “place” was used. Martin found among the Chinantec and Mixe people a similar phrase (*kam*), but for habitats named after the dominant species cf. [10]. “Locative-like verbal expressions” were also used by Csángós (e.g. areas trampled (or not) by animals or humans, Table 6 cf. [11,13]).

In some cases, different people described the habitat of a particular plant species by using different habitat terms. In these cases, the exact meaning of the terms used was often not the same, as they referred to a different (though often overlapping) section of the same environmental gradient, or described the habitat by using another environmental feature. Shepard et al. [11] also documents such examples in the Matsigenka folk classification (“apamankera nia – place of inundation, flooding” and “otsegoa – seasonally flooded island, branch of river”), though without a detailed discussion. A typical example from Gyimes was the terms *porond* (gravel deposit) and *vízmeghajtotta hely* (an area often flooded, with erosion and accummulation of gravel and sand). The former was the prototype, emphasizing that the area is elevated, whereas the latter mostly referred also to such places, but emphasized disturbance. The latter and its variants were used 14 times, of which 8 described habitats of *Myricaria germanica* and bushy *Salix* spp., species typical of gravel deposits (*porond*s).

Habitats that the studied community hardly knows may also represent important data in terms of folk landscape partitioning [6]. Csángós did not care much about rocky habitats and habitats of the extremely rare aquatic vegetation, and did not consider the differences caused by the heterogenous spatial pattern of co-dominant tall grasses. The former two were insignificant in their economy, whereas the dominant grasses formed a functionally uniform group (broad-leaved, tall grasses) whose division into any further subgroups might have seemed meaningless.

While some geomorphological features were also used as a habitat term (e.g. *völgy* (valley), *tető* (mountain top), *oldal* (mountain slope)), others were never mentioned as a habitat (e.g. *bitkó* (a small peak), *nyak* (neck), *hegyláb* (foot hill)). It seems that the former three have a special combination of environmental features, while the others have not.

Compared to the scientific botanical classification [39], the Csángó system was only less detailed in the case of fen communities (communities dominated by *Eriophorum sp.*, *Glyceria plicata*, *Carex* spp.) and rocky vegetation. In all other cases, the traditional landscape partitioning was more detailed than the scientific classification [cf. 39].

### Scale

A special dimension in Csángó landscape partitioning is scale. Scale has two aspects: (1) topographical (spatial scale): some habitat categories (e.g. high mountains, settlements) occupy larger areas often comprising a habitat mosaic of finer-scaled habitats, while others extend only to smaller areas (e.g. stream side, birch woodland), and (2) topological scale: some more inclusive habitat categories (e.g. forest) are subdivided into subcategories (e.g. into spruce and beech forest, dense or sparse forest etc.), while others are not (e.g. gravel deposit, *Nardus* grassland). The two aspects are not independent. More inclusive categories often cover larger territories, while less inclusive ones usually cover smaller areas (cf. to different ranks in folk biological species classifications [cf. 1], and in landscape ethnoecological partitionings e.g. [11,18,54]). Gilmore et al. [21] for example identified three main categories in his general group of habitats: (1) “cuadu” (lit. soft earth) for the swamp habitats, (2) “aqui” (ugly forest) and (3) habitats without soft earth and ugly forest. Shepard et al. [11] also lists inclusive categories. The Matsigenka *nigankipatsa* (lit. ‘middle earth’) is a broad category for habitats that are not flooded, but *nigankipatsa* is not included into any specific biotic/abiotic habitat type (other inclusive habitats: ‘flat area’, ‘many hills’ (= montane vegetation, above 600 m) [11].

Habitats that Csángós distinguished could be arranged into characteristic categories at three spatial scales: macro-, meso-, and microhabitats. Similarly to Shepard et al. [11], Abraão et al. [18], and Gilmore et al. [21], we also found that abiotic features (e.g. geomorphology, hydrology, edaphic conditions) often defined larger, broader habitat categories, while biotic feature-defined habitats used in finer partitioning. Habitats in the land-use set were often intermediate between macro and meso scales. Habitats elicited by Shepard et al. [11] are mostly mesohabitats. Microhabitats are not mentioned. Martin [10] found that the Chinantec have a keen sense of microclimate. They distinguish sites with a special environment (i.e. microhabitats) that can be used for cultivation of certain plants outside of their zonal range.

In conclusion, macrohabitats occupy usually large areas and comprise many habitat types, mesohabitats are usually smaller in extension and homogenous and are often dominated by one vegetation type, while microhabitats are embedded in mesohabitats and provide special niches for particular species. One reason for the use of different scales in landscape partitioning may be ecological, since species occupy somewhat different places (niches) in a landscape, some species are specialist, others generalist. For a precise description of these species-specific habitats a multi-scaled landscape partitioning might be better suited.

### Indicator species

Csángós never used species composition (list of characteristic and/or dominant species) as one of their features, although species composition is the key feature in recognizing and classifying habitats for scientific purposes see e.g. [34,39]. Even when they were asked to list the typical species of a particular habitat, they listed only a few species (Babai & Molnár unpubl.). We emphasize that the many habitat terms derived from a species name (Table 3) did not reflect a scientific plant community used by phytosociologists and as suggested by Rab [7] in the nearby Gyergyó-basin, but only pointed to the most salient species of that spot in case of the Csángós.

Indicator species, however, were often used by Csángós in describing plant species’ habitats. These indicator species were not necessarily the commonest ones in a given habitat, but were morphologically or ecologically salient see also [18,20,25]. Whereas the majority of indicator species are woody in the communities studied elsewhere [18,35], the indicator species were primarily herbaceous plants in Gyimes.

Species regarded as indicators were mainly very well known in the community, and their habitat preferences were widely understood (Babai & Molnár unpubl.). These features made them well suited to characterize the habitat preferences of other species. The majority of indicator species were ecological specialists in this landscape, found mostly in a single habitat type (e.g. *Onobrychis viciifolia, Nardus stricta, Leontopodium alpinum, Tragopogon orientalis*). Surprisingly, the list also included some generalist species (e.g. *Picea abies*) that, however, were used to describe the habitats of other generalist species (e.g. *Leucanthemum vulgare*, *Abies alba*).

Some meadow types were named after their typical indicator species (Table 12). These species were generally not dominant, but were rather associated with certain characteristics of the habitat, most often with the treatment with manure. Species indicating manured sites were *Tragopogon orientali*s and *Salvia pratensis*. *Nardus stricta* was an indicator species of nutrient poor, acidic habitats at higher elevations. *Onobrychis* indicated nutrient poor, south-facing slopes. *Pteridium aquilinum* may become abundant in south-facing and rather dry slopes, whereas *Alchemilla* spp. reached high abundances at higher elevations, primarily in grazed grasslands. Among the Chinantec and Mixe, Martin [10] also documented indicator species that are used to indicate soil types suitable or not for the cultivation of certain plants.

### Multidimensionality of the Csángó landscape partitioning

Csángó landscape ethnoecological partitioning was multidimensional, as it incorporated several sets of features. Multidimensional landscape partitioning was suggested as a general phenomenon by Ellen [20] and Hunn and Meilleur [22], and was found among Matsigenka in Peru by Shepard et al. [11], various First Nations groups in British Columbia [26], and Fulani pastoralists in Burkina Faso [25]. The number of gradients are usually large in mountainous landscapes, but Csángós have also contributed to an increased number of gradients (or an increased expression of the existing gradients, e.g. on south facing slopes, or along the valley bottoms) by turning the forest-dominated landscape into a more diverse mosaic of grasslands, forests, and arable fields cf. [3,4]. This resulted in an increased number of combinations of environmental features that plant species can inhabit. Csángó people used these combinations to describe, and name habitats of plant species they use as a resource.

Where there is a single dimension of morphological discontinuity, a salient prototype can generate a one dimensional classification of related forms [1], but the multidimensional nature of habitat dictates that classification too will likely be multidimensional [22]. In Gyimes, several gradients could be recognized in the study area, which served as the basis of multidimensional landscape partitioning (i.e. mountain top / mountain side-valley, forest / bush / grassland, rocky / poor / lush, wet / moist / dry). These gradients were often formed from simple dichotomous pairs cf. [11], but more stages may have been included between the extremes in the classification of a rather complicated habitat mosaic. Besides land-use types, these antagonistic pairs formed the basis for the basic-level landscape partitioning, and helped recognize the most salient habitats in the landscape cf. [22]. Environmental features were combined flexibly in habitat descriptions see also [4,11,13,15,35].

In South and Central America, South East Asia, and Canada, features used in multidimensional landscape partitioning are almost the same as those in Gyimes e.g. [10,11,13,20,26]. Most of them also appear in Europe (e.g. hydrological, geomorphological, vegetational features) [3,4,54]. The most striking exceptions are the successional stages of the swidden agricultural systems, as these agro-forestry systems were banned in Europe by the German-type forest administration during the 19^th^ and early 20^th^ centuries [56].

On the contrary, Molnár [54] found that herders’ habitat classification in a salt steppe environment (Hortobágy, Hungary) is not multidimensional. He assumed that in the flat, open, and on the medium- and long-term stable landscape, the various abiotic and biotic factors that determine different habitats are arranged along a single (key) gradient (depth of ground-water table), while many possible gradients (e.g. woody / non-woody, mountain / valley, rock / sand, successional, naturalness) are missing. Among the Baniwa people in Amazonia, Abraão et al. [18] also documented a landscape partitioning with only one gradient. The authors could structure forests along this gradient into a single hierarchical system (with 5 ranks) analogous to Berlin’s [1] folk biological classification system.

Based on these experiences, we propose the following hypothesis for testing: multidimensionality of landscape partitionings and the number of dimensions applied depend on the number of key environmental gradients in a given landscape. Hunn and Meilleur [22] argue that folk landscape ethnoecological partitionings are organized into shallow classifications only. Ellen [20] goes further by arguing that multidimensionality and the emic multidimensional continuum is what might prevent the development of a single well developed hierarchy. Gilmore et al. [21] found that folk habitat types are grouped into several, separate, overlapping subsystems. Further detailed studies are needed that document emic classifications of habitats to deepen our knowledge on the relations of multidimensionality and hierarchy.

In conclusion we argue that multidimensional partitioning of the landscape in Gyimes made the nuanced characterization of plant species’ habitats possible. Multidimensional partitioning provided innumerable possibilities for the Csángós to combine the most characteristic and salient features when explaining the habitat preference of a particular wild plant species in their diverse, semi-natural, and intensively managed landscape. The number of dimensions applied by them seemed to depend on the number of key environmental gradients in their mountainous landscape.

## End notes

^a^ Vegetation can be classified in many ways. Methodologies are based on species composition, vegetation physiognomy, vegetation structure, or environmental factors. For fine-scale surveys, floristic approaches are the best suited. In the so-called Braun-Blanquet floristic-sociological approach, plant community types are conceived as units recognized by their total floristic composition. For a so-called phytosociological survey, relevees are taken, usually several by several meters quadrats. Beside the list, the dominance (cover) of all plant species is given in percent or in an ordinary scale, 1–5. Relevees are subsequently arranged according to floristic similarity. Plant associations are delimited based on characteristic species (species specific to that association), differential and dominant species. With the plant associations described in a region, actual and potential vegetation of the area can be mapped. Plant associations are usually named by using a characteristic and a dominant species. Author(s) of the associations are also given.

## Competing interests

The authors declare that they have no competing interests.

## Authors’ contributions

Both authors made substantial contributions to conception and design. DB carried out most of the data collection and analysis. Both authors were involved in drafting and writing the manuscript. Both authors read and approved the final manuscript.

## References

[B1] Berlin B Ethnobiological Classification. Principles of Categorisation of Plants and Animals in Traditional Societies 1992 Princeton: Princeton University Press

[B2] Johnson LM Hunn ES Johnson LM, Hunn ES Introduction Landscape Ethnoecology. Concepts of Biotic and Physical Space 2010 New York, Oxford: Berghahn Books 1 11

[B3] Meilleur B Alluetain Ethnoecology and Traditional Economy: The Procurement and Production of Plant Resources in the Northern French Alps 1986 PhD thesis, Washington: University of Washington

[B4] Meilleur B Johnson LM, Hunn ES The structure and Role of Folk Ecological Knowledge in Les Allues, Savoie (France) Landscape Ethnoecology. Concepts of Biotic and Physical Space 2010 New York, Oxford: Berghahn Books 159 174

[B5] Molnár Z Traditional ecological knowledge of herders on the flora and vegetation of the Hortobágy 2012 Debrecen: Hortobágy Természetvédelmi Közalapítvány

[B6] Péntek J Szabó TA Ember és növényvilág. Kalotaszeg növényzete és népi növényismerete[People and plantlife. The vegetation and folk plant knowledge of Kalotaszeg.] 1985 Bukarest: Kriterion Könyvkiadó

[B7] Rab J Népi növényismeret a Gyergyói-medencében. [Folk plant knowledge in the Gyergyó Basin.] 2001 Csíkszereda: Pallas-Akadémia Könyvkiadó

[B8] Rotherham ID The Implications of Perceptions and Cultural Knowledge Loss for the Management of Wooded Landscapes: A UK case-study Forest Ecol 2007 249 100 115 10.1016/j.foreco.2007.05.030

[B9] Nch I-W Hunn SE with J Selam and family The big river”. Mid-Columbia indians and their land 1990 Seattle and London: University of Washington Press

[B10] Martin GJ Ecological classification among the Chinantec and Mixe of Oaxaca, Mexico Etnoecológica 1993 1 17 33

[B11] Shepard G Yu DW Lizarralde M Italiano M Rain Forest Habitat Classification among the Matsigenka of the Peruvian Amazon J 2001 21 1 38

[B12] Torre-Cuadros MA Ross N Secondary Biodiversity: Local Perceptions of Forest Habitats, the Case of Solferino, Quintana Roo, Mexico J 2003 23 287 308

[B13] Johnson LM A Place That’s Good. Gitksan Landscape Perception and Ethnoecology Hum Ecol 2000 28 2 301 325 10.1023/A:1007076221799

[B14] Verlinden A Dayot B A comparison between indigenous environmental knowledge and a conventional vegetation analysis in north central Namibia J Arid Environ 2005 62 143 175 10.1016/j.jaridenv.2004.11.004

[B15] Halme KJ Bodmer RE Correspondence between Scientific and Traditional Ecological Knowledge: Rain Forest Classification by the Non-indigenous Riberenos in Peruvian Amazonia Biodivers Conserv 2007 16 1785 1801 10.1007/s10531-006-9071-4

[B16] Hernandez-Stefanoni JL Pineda JB Valdes V Comparing the use of indigenous knowledge with classification and ordination techniques for assessing the species composition and structure of vegetation in a tropical forest Environ 2006 37 686 702 10.1007/s00267-004-0371-8 16508801

[B17] Roturier S Roué M Of forest, snow and lichen: Sámi reindeer herders’ knowledge of winter pastures in northern Sweden Forest Ecol 2009 258 960 967

[B18] Abraão MB Shepard GH Nelson BW Jr Baniwa JC Andrello G Yu DW Johnson LM, Hunn ES Baniwa Vegetation Classification in the White-Sand Campinarana Habitat of the Northwest Amazon, Brazil Landscape Ethnoecology. Concepts of Biotic and Physical Space 2010 New York, Oxford: Berghahn Books 83 115

[B19] Davidson-Hunt I Berkes F Johnson LM, Hunn ES Journeying and Remembering: Anishinaabe Landscape Ethnoecology from Northwestern Ontario Landscape Ethnoecology. Concepts of Biotic and Physical Space 2010 New York, Oxford: Berghahn Books 222 240

[B20] Ellen R Johnson LM, Hunn ES Why aren’t the Nuaulu like the Matsigenka? Knowledge and Categorization of Forest Diversity on Seram, Eastern Indonesia Landscape Ethnoecology. Concepts of Biotic and Physical Space 2010 New York, Oxford: Berghahn Books 116 140

[B21] Gilmore MP Ochoa SR Flores SR Johnson LM, Hunn ES The Cultural Siginicance of the Habitat Maňaco Taco to the Maijuna of the Peruvian Amazon Landscape Ethnoecology. Concepts of Biotic and Physical Space 2010 New York, Oxford: Berghahn Books 141 158

[B22] Hunn ES Meilleur BA Johnson LM, Hunn ES Toward a Theory of Landscape Ethnoecological Classification Landscape Ethnoecology. Concepts of Biotic and Physical Space 2010 New York, Oxford: Berghahn Books 15 26

[B23] Johnson LM Hunn ES Landscape ethnoecology. Concepts of Biotic and Physical Space 2010 New York, Oxford: Berghahn Books

[B24] Mark DM Turk AG Stea D Johnson LM, Hunn ES Ethnophysiography of Arid Lands: Categories for Landscape Features Landscape Ethnoecology. Concepts of Biotic and Physical Space 2010 New York, Oxford: Berghahn Books 27 48

[B25] Krohmer J Johnson LM, Hunn ES Landscape Perception, Classification, and Use among Sahelian Fulani in Burkina Faso Landscape Ethnoecology. Concepts of Biotic and Physical Space 2010 New York, Oxford: Berghahn Books 49 82

[B26] Johnson LM Trail of Story, Traveller’s Path. Reflections on Ethnoecology and Landscape 2010 Athabasca: AU Press, Athabasca University

[B27] Hájková P Roleček J Hájek M Horsák M Fajmon K Polák M Jamrichová E Prehistoric origin of the extremly species-rich semi-dry grasslands int he Bílé Karpaty Mts (Czech Republic and Slovakia) Preslia 2011 83 185 204

[B28] Tasser E Tappeiner U Impact of land use changes on mountain vegetation Appl 2002 5 173 184 10.1111/j.1654-109X.2002.tb00547.x

[B29] Berkes F Colding J Folke C Rediscovery of Traditional Ecological Knowledge as Adaptive Management Ecol Appl 2000 10 1251 1262 10.1890/1051-0761(2000)010[1251:ROTEKA]2.0.CO;2

[B30] Menzies CR Butler C Menzies CR Introduction. Understanding Ecological Knowledge Traditional Ecological Knowledge and Natural Resource Management 2006 Lincoln and London: University of Nebraska Press 1 17

[B31] Berkes F Sacred Ecology: Traditional Ecological Knowledge and Resource Management 1999 Philadelphia: Taylor & Francis

[B32] Brown J Mitchell N Beresford M Brown J, Mitchell N, Beresford M Preface The protected landscape approach. Linking Nature, Culture and Community 2005 Gland (Switzerland) and Cambridge: IUCN 9 10

[B33] Molnár Z Bartha S Babai D Szabó P, Hedl R Traditional ecological knowledge as a concept and data source for historical ecology, vegetation science and conservation biology: A Hungarian perspective Human Nature. Studies in Historical Ecology and Environmental History 2008 Brno: Institute of Botany of the ASCR 14 27

[B34] Borhidi A Magyaroszág növénytársulásai. [Plant associations of Hungary.] 2003 Budapest: Akadémiai Kiadó

[B35] Fleck DW Harder JD Matses indian rainforest habitat classification and mammalian diversity in amazonian Peru J 2000 20 1 36

[B36] Babai D Molnár Z Népi növényzetismeret Gyimesben II.: Termőhely- és élőhelyismeret. [Traditional ecological knowledge in Gyimes II.: Knowledge on habitats.] Bot. Közlem 2009 96 145 173

[B37] Dobos F A Gyimesi-szoros földrajza. [The geography of the Gyimes-strait.]Geographica Pannonica 33 1939 Pécs: Kultúra nyomda

[B38] Pálfalvi P A Gyimesi-hágó (1164 m) környékének florisztikai vázlata [Floristic sketch of the surroundings of the Gyimes Pass (1164 m).] Múzeumi Füzetek (Az Erdélyi Múzeum Egyesület Természettudományi és Matematikai Szakosztályának Közleményei) 1995 4 107 114

[B39] Nechita N Flora şi vegetaţia cormofitelor din Masivul Hăşmas, Cheile Bicazului şi Lacu Roşu 2003 Piatra-Neamţ: Muzeul de Ştiinţe Naturale

[B40] Ilyés Z A tájhasználat változásai és a történeti kultúrtáj 18–20. századi fejlődése Gyimesben. [Landscape changes and the 18–20. century development of the historical cultural landscape in Gyimes.] 2007 Eger: Eszterházy Károly Főiskola

[B41] Hofer T Hofer T A gyimesi csángó népcsoport kialakulása. [Origin of the Gyimes Csángó ethnic group.] Antropológia és/vagy néprajz. Tanulmányok két kutatási terület vitatott határvidékéről. [Antropology and/or ethnography. Studies from the border region of two disciplines] 2009 Budapest: L’Harmattan Kiadó 66 77

[B42] Babai D Magashegyi növényzet etnoökológiai értékelése a Keleti-Kárpátokban (Gyimes). (Ethnoecological evaluation of mountain vegetation in the Eastern Carpathians (Gyimes) 2012 University of Pécs: PhD Dissertation

[B43] Kallós Z Gyimesvölgyi keservesek [Said songs from Gyimes] Néprajzi Közlem 1960 5 3 51

[B44] Tánczos V Zakariás E, Keszeg V Gyimesi archaikus népi imádságok és ráolvasások. [Archaic prayers and incantations in Ghimes region.] A Kriza János Néprajzi Társaság Évkönyve 2. [Annals of the Kriza János Ethnographic Society. 2.] 1994 Kolozsvár: Kriza János Néprajzi Társaság 211 243

[B45] Pócs E Pócs E **Előszó.** [**Foreword.**] Vannak csodák, csak észre kell venni. Helyi vallás, néphit és vallásos folklór Gyimesben I. [There are wondres, if you see them. Local religion, folk beliefs and religious folkore in Gyimes] 2008 Budapest: L’Harmattan Kiadó 7 14

[B46] Sólyom A Knowles B Bogdán J Rodics G Biró R Nyírő G Small scale farming in the Pogány-havas Region of Transylvania. Farming statistics, agricultural subsidies, the future of farming. Final Report 2011 Miercurea Ciuc: Pagan Snow Cap association 97

[B47] Bíró R Demeter L Knowles B Knowles B Farming and management of hay meadows in Csík and Gyimes – Experiences from social research Mountain hay meadows. Hotspots of biodiversity and traditional culture 2012 http://mountainhaymeadows.eu/online_publication/11-farming-and-management-of-hay-meadows-in-csik-and-gyimes.htmlLast download: on the 27^th^ October 2012

[B48] Krausmann F Milk, Manure and Muscle Power. Livestock and the Transformation of Preindustrial Agriculture in Central Europe Hum Ecol 2004 32 735 772 10.1007/s10745-004-6834-y

[B49] Molnár Z Babai D Népi növényzetismeret Gyimesben I.: Növénynevek, népi taxonómia, az egyéni és közösségi növényismeret. [Folk plant knowledge in Gyimes I.: Plant names, folk taxonomy, plant knowledge on individual and community level.] Bot. Közlem 2009 96 1–2 117 143

[B50] Puri RK Watson CW Newing, H Conducting Research in Conservation. A social science perspective 2011 London and New York: Routledge Taylor & Francis Group 376

[B51] Grey-Wilson C Blamey M Pareys Bergblumenbuch: wildblühende Pflanzen der Alpen, Pyrenäen, Apenninen, der skandinavischen und britischen Gebirge 1980 Hamburg, Berlin: Parey

[B52] Ellen R The cultural relations of classification: an analysis of Nuaulu animal categories from central Seram 1993 Cambridge: Cambridge University Press 315

[B53] Hunn E Toward a perceptual model of folk biological classification American Ethnologist 1976 3 3 508 524 10.1525/ae.1976.3.3.02a00080

[B54] Molnár Z Classification of Pasture Habitats by Hungarian Herders in a Steppe Landscape (Hungary) 2012 Ethnomed: J. Ethnobiol (accepted) 10.1186/1746-4269-8-28 PMC3533854 22853549

[B55] Netting RMC Balancing on an Alp. Ecological change & continuity in a Swiss mountain community 1981 Cambridge: Cambridge University Press

[B56] Johann E Agnoletti M Bölöni J Erol SC Holl K Kusmin J Latorre JG Molnár Z Rochel X Rotherham ID Saratsi E Smith M Tarang L Benthem M Laar J Parotta JA, Trosper RL Europe Traditional Forest-Related Knowledge Sustaining Communities, Ecosystems and Biocultural Diversity, World Forests 12 2012 Dordrecht, Heidelberg, London, New York: Springer Science + Business Media B. V 203 249

